# Artificial intelligence framework for heart disease classification from audio signals

**DOI:** 10.1038/s41598-024-53778-7

**Published:** 2024-02-07

**Authors:** Sidra Abbas, Stephen Ojo, Abdullah Al Hejaili, Gabriel Avelino Sampedro, Ahmad Almadhor, Monji Mohamed Zaidi, Natalia Kryvinska

**Affiliations:** 1 Department of Computer Science, COMSATS University Islamabad, Islamabad, Pakistan; 2 Department of Electrical and Computer Engineering, College of Engineering Anderson, Anderson, SC 29621 USA; 3 Computer Science Department, Faculty of Computers and Information Technology, University of Tabuk, Tabuk, 71491 Saudi Arabia; 4 Faculty of Information and Communication Studies, University of the Philippines Open University, Los Baños, 4031 Philippines; 5 Center for Computational Imaging and Visual Innovations, De La Salle University, 2401 Taft Ave., Malate, 1004 Manila Philippines; 6 Department of Computer Engineering and Networks, College of Computer and Information Sciences, Jouf University, 72388 Sakaka, Saudi Arabia; 7 Department of Electrical Engineering, College of Engineering, King Khalid University, Abha, Saudi Arabia; 8 Information Systems Department, Faculty of Management, Comenius University in Bratislava, Odbojárov 10, 82005 Bratislava 25, Slovakia

**Keywords:** Mathematics and computing, Computer science, Information technology

## Abstract

As cardiovascular disorders are prevalent, there is a growing demand for reliable and precise diagnostic methods within this domain. Audio signal-based heart disease detection is a promising area of research that leverages sound signals generated by the heart to identify and diagnose cardiovascular disorders. Machine learning (ML) and deep learning (DL) techniques are pivotal in classifying and identifying heart disease from audio signals. This study investigates ML and DL techniques to detect heart disease by analyzing noisy sound signals. This study employed two subsets of datasets from the PASCAL CHALLENGE having real heart audios. The research process and visually depict signals using spectrograms and Mel-Frequency Cepstral Coefficients (MFCCs). We employ data augmentation to improve the model’s performance by introducing synthetic noise to the heart sound signals. In addition, a feature ensembler is developed to integrate various audio feature extraction techniques. Several machine learning and deep learning classifiers are utilized for heart disease detection. Among the numerous models studied and previous study findings, the multilayer perceptron model performed best, with an accuracy rate of 95.65%. This study demonstrates the potential of this methodology in accurately detecting heart disease from sound signals. These findings present promising opportunities for enhancing medical diagnosis and patient care.

## Introduction

The heart is the body’s most important organ, pumping blood to all the other tissues and organs. However, it is also vulnerable to disease and trauma, negatively impacting human health and leading to heart-related ailments collectively known as “Cardiovascular Disease (CVD)”^1^. When risk factors, including high cholesterol, smoking, inactivity, and hypertension, are present, they can lead to trouble breathing, weakness, exhaustion, and more. In 2016, CVD was responsible for an estimated 17.9 million individuals worldwide, or 31% of all deaths^2^. Unfortunately, over 70% of these deaths were attributable to CVD, concentrated in low and middle-income nations. However, it should be stressed that many of these diseases can be avoided via precaution, with early detection being a key factor.

The heartbeat of a healthy human heart exhibits a predictable rhythm due to the regular opening and closing of the heart’s valves. Murmur is an example of an anomaly since it deviates from the usual. Although cardiac murmurs are not usually dangerous, they can signify various potentially life-threatening heart conditions^3^. Expert doctors can hear murmurs from a mile away but may not always be accessible, especially in rural areas where primary care physicians are scarce. A cardiac murmur might be an important indicator of cardiovascular disease in its early stages. Skilled doctors and physicians can usually discover this irregular sound pattern, which is often an indicator of underlying cardiac abnormalities, by a method called Cardiovascular auscultation, in which they listen to the heart’s noises with a stethoscope^4^.

Early detection and accurate prediction are crucial to treat and lessen cardiovascular disease’s deadly effects effectively. Medical experts widely use angiography for diagnosis, but its lengthy and costly nature presents difficulties, especially in countries with low resources^5^. Machine learning and artificial intelligence are two emerging technologies that potentially improve the health outcomes of those at risk for cardiovascular illnesses^6,7^. There has been a significant increase in the use of ML and DL in medical diagnostics, which helps with disease classification and identification and gives clinicians more information to diagnose better and treat patients^6–9^. The application of ML to the field of medical diagnostics has been gaining traction recently, and for good reason^10^. Improvements in disease diagnosis, treatment, and prevention have resulted from the accumulation of data made possible by recent advances in disease classification and identification. ML and DL methods have proven crucial in determining how often certain diseases occur.

### Research gap

There is a rising need for accurate and reliable methods when diagnosing cardiovascular diseases. Early diagnosis is crucial for improving patient outcomes due to the prevalence of CVD worldwide. One potentially effective approach to identifying cardiac illness is using acoustic waves. Heart sounds are the audible vibrations the heart muscle produces when it contracts rhythmically. Heart valve disease, heart failure, and coronary artery disease are just a few examples of the many cardiac conditions that can be diagnosed using a variety of acoustic signals. The use of ML and DL techniques has been successful in classifying heart sounds. However, there are several gaps in this area of study^8,11^. The need for broad and diverse datasets is a key subject for future study. The datasets used for heart sound classification often have size and fairness issues. Models suffering from poor generalizability to real-world datasets may be the outcome of this phenomenon. The need for stronger and more efficient ML and DL models is another hotspot for investigation. Current methods for classifying heart sounds are computationally expensive and time-consuming to train. This can create difficulties when putting these concepts into practice. The proposed research utilizes a large and varied heart sound dataset to close current knowledge gaps. Novel ML and DL models with improved robustness and efficiency compared to existing models will also be developed as part of this study. The results of the planned study will be very helpful in developing more accurate and reliable methods for diagnosing cardiovascular diseases. This measure can potentially improve patient outcomes and lessen the impact of cardiovascular disease. The study’s projected findings have the potential to make important advancements in the identification of cardiac disease. Researchers can now tap into a large and varied database of cardiac recordings. Developments in the Future Novel models with improved robustness and efficiency will likely be developed thanks to ML and DL. The accuracy of ML and DL models for classifying heart sounds should be improved.

*Research Contribution:* The main contributions of this research are:

- Propose a novel approach that integrates cutting-edge techniques such as audio data augmentation with machine learning (ML) and deep learning (DL) methodologies to enhance and optimize the detection rate of heart disease through the synergistic application of these advanced techniques.
- Utilize two subsets of the PASCAL challenge dataset, created a new database of noisy heart sound signals and employed the proposed approach to diagnose heart disease from noisy audio signals.
- Develop a feature ensembler by combining multiple audio feature extraction methods to improve the performance of the ML and DL models.
- Utilize multiple ML and DL models, provide comparison with previous studies and obtain significant improvement in heart disease detection rate.

### Research organization

This research describes a novel approach for detecting heart disease from sound signals using machine learning and deep learning techniques in this study. Section “Literature Review” describes the literature analysis to evaluate existing methodologies, noting their strengths and weaknesses. Our suggested method uses a carefully selected dataset and noise induction to replicate real-world circumstances, presented in the Section “Dataset Selection”. The complete methodology of this research is described in the Section “Proposed Methodology”. The experimental findings and discussions in the section “Experimental Results and Discussion” show that our model is effective, encompassing performance parameters like accuracy, precision, recall, and F1-score. We compare our findings to those of previous studies to demonstrate the superiority of our technique in predicting heart disease, shown in the subsection “Comparison With Existing Studies”. Finally, this research emphasizes the importance of ML-based detection and details prospective future work for further development and its potential influence on healthcare in the section “Conclusion”.

## Literature review

In this section, this research discussed the methods used before to identify CVD from audio signals. Classifying heart sounds typically involves three steps: segmenting the heart sounds, extracting features from the heart sounds, and finally, classifying the heart sounds. The first step is to pinpoint the precise position of the heart’s fundamental rhythmic sounds. Each Phono Cardiography (PCG) recording extracts multiple heart disease sounds. Accurately identifying the heart sounds provides insight into the heart’s systolic and diastolic sounds. Since the primary objective of aberrant heart sound detection is abnormality identification rather than detection, segmentation is unnecessary. As a result, many approaches have been proposed in the literature for classifying heart sounds without resorting to segmentation. Comparable outcomes are possible when combining segmentation data from different approaches. Table 1 summarizes the results of previous efforts to classify heart diseases.

Several researchers have looked into using Artificial Intelligence (AI) for cardiac disease classification, and the results have been promising^12,13^. Machine learning models have successfully diagnosed heart problems using diverse data modalities such as electrocardiograms and imaging. Recent advances in audio signal analysis have opened up a new research field. While preliminary research indicates that audio-based AI models can reach high accuracy in cardiac disease categorization, it is crucial to emphasize that this is a very new and expanding field of study. As a result, the accuracy of audio-based AI frameworks should be evaluated with caution, and larger, more robust investigations are required to validate their accuracy and therapeutic value^14,15^.

Numerous feature extraction techniques have been discussed in the literature, and they can be roughly classified into one of three broad classes: time domain, frequency domain, or time–frequency complexity domain^16–18^. The physiological characteristics of PCG signals make the time or frequency domain features intuitive, easy to understand, and straightforward to compute. However, independently quantifying essential information in PCG signals in the time and frequency domains can be difficult. This has led to the popularity of extracting features in the Time–Frequency (TF) domain. TF-based features can provide more in-depth information about the PCG signal and better feature extraction performance outcomes, but they are more computationally intensive to generate^19^. Wavelet transforms, Discrete and Packet Wavelet Transforms (DPWT), Hilbert transforms, Empirical Wavelet Transforms (EWT), Variational Mode Decomposition (VMD), and Tunable Q-Wavelet Transforms (TQWT) are all common ways to extract TF features from PCG data. Using spine CT to create the PCG signal’s TF matrix improves both its sensitivity to pathological alterations and its ability to focus on the TF domain. However, feature extraction is still challenging because PCG signals are non-stationary and have many characteristics.

The last step is to use the recovered features to train a classifier to predict each PCG signal^12,13^. Several machine learning-based classifiers have been proposed to classify heart sounds based on extracted features. The performance of the classifiers was further improved by using an ensemble of classifiers. Tent-pooling decomposition and a graph-based feature generator are proposed by the authors of research^20^ for feature extraction. DT, linear discriminant, and Support Vector Machine (SVM) models were used to classify PCG signals into five groups after features were defined using iterative Neighborhood Component Analysis (NCA). The authors of research^21^ chose the most distinguishing features for NCA using a 1D-binary pattern with three kernels. Classification of PCG signals was accomplished using KNN and SVM. Six audio variables were collected from PCG signal audio samples and categorized using four conventional machine learning-based classifiers (zero crossing rate, energy entropy, volume, spectral flux, spectral centroid, and spectral roll-off)^22^. Categorizing PCGs is still subjective and time-consuming, even though ML-based algorithms have made great strides in this area. Several deep learning models, including Convolutional Neural Networks (CNN) and LSTMs, have recently been used to classify heart sounds^23^. Their ability to automatically analyze heart sounds and extract high-level representations has garnered increasing attention. The practice of identifying PCG signals from whole audio recordings rather than from smaller segments is also gaining traction. Authors in^24^ used digitally recorded stethoscope audio waves to create phonocardiograms (PCGs) for heart disease detection. PCG signals are classified into five categories using deep learning models, and spectrograms are processed using a Regularised Convolutional Neural Network. The model achieves 94% accuracy in a Python simulation environment. The study develops a decision support system for remote heart state assessment in response to the importance of early identification in the fight against cardiovascular disease. Open-access Kaggle datasets from the PASCAL heart sound categorization challenge are used for training and testing. While the study intends to use ECG and EEG signals in the future to increase accuracy, it does not address real-world clinical applicability, data quality, or noise difficulties in audio recordings.

By conducting an extensive investigation using various acoustic feature aggregation and data augmentation strategies^25^, tackled the difficult task of ambient sound classification (ESC). Various audio feature extraction techniques are utilized in the suggested data augmentation methods, emphasizing spectrogram image features (SIFs) that are reinforced, aggregated, and combined. The logarithmic scale of the Mel spectrogram is used to introduce two new characteristics, L2M and L3M. Two approaches, NA-1 and NA-2, are born from integrating these features with Mel and LM. NA-2 requires the vertical aggregation of these images in pairs, whereas NA-1 improves SIF data by integrating different audio features based on spectrograms. Three popular ESC benchmark datasets-ESC-10, ESC-50, and Urbansound8k (Us8k)-train the transfer learning model DenseNet-161, which was fine-tuned with individual optimal learning rates using discriminative learning approaches. As a pre-processing technique, quiet cutting is used since many audio clips contain silent parts. This approach provides state-of-the-art results on all ESC datasets, with Us8k achieving 97.98% accuracy, ESC-50 98.52%, and ESC-10 99.22%. We further evaluate these approaches on real-time audio data and show they perform competitively. Among the most notable additions are the revolutionary NA-1 and NA-2 procedures, surpassing previous methods and including L2M and L3M features.

Authors in^26^ included the difficulties encountered in ambient sound classification (ESC), sometimes called Sound Event Recognition (SER), as a result of variables such as different frameworks, overlapping sound events, numerous sound sources, and non-uniform distances between microphones and acoustic sources. The fast integration of ESC tasks into many everyday contexts encourages the search for efficient approaches. To improve the performance of ESC tasks, the study uses deep convolutional neural networks (DCNN) that have been trained using regularisation and data improvement using fundamental audio properties. The performance of two deep convolutional neural network (DCNN) models is compared: Model-2 without max-pooling and Model-1 with max-pooling. We take a look at three benchmark datasets-ESC-10, ESC-50, and Urban sound (US8K)-and three methods for extracting audio features: Mel spectrogram (Mel), Mel frequency cepstral coefficient (MFCC), and Log-Mel. Combining L2 regularisation with the original datasets, the paper presents offline data augmentation approaches to decrease the risk of overfitting caused by restricted data. On supplemented datasets, the DCNN (Model-2) achieves the best accuracy, which does not use max-pooling and uses Log-Mel for audio feature extraction. The achieved accuracies are 94.94% for ESC-10, 89.28% for ESC-50, and 95.37% for US8K. Environmental sound categorization difficulties are where the suggested method shines, according to the results of the experiments. The experimental study aims to investigate the use of convolutional neural networks (CNNs) for ESC tasks, specifically looking at two stacked DCNN models. Although Model-2 does not use max-pooling, Model-1 does. Both models are evaluated on real audio datasets using three different feature extraction methods (Mel, MFCC, and Log-Mel). Datasets that are supplemented offline follow the identical experimental protocol. The study demonstrates that DCNN models are useful; however, Model-2 and Log-Mel extraction stand out for their exceptional accuracy rates on various ESC datasets.

An important bioelectrical indication during muscle contraction, surface electromyography (sEMG) signals are the subject of research’s classification efforts, with a focus on their potential usefulness in controlling prosthetic limbs for the upper limbs^27^. They used an E2CNN, an efficient concatenated convolutional neural network optimized for fast response and real-time performance, to achieve these goals. This work tests the model on two datasets: the publicly accessible NinaPro DB1 dataset and a longitudinal dataset with ten non-disabled and six trans-radial amputee individuals across seven days of data collection. Preprocessing converts the raw sEMG signals into LMSs or Log-Mel spectrograms. Concatenation layers, unique to the E2CNN design, merge input layers with the output of every convolutional block. On the longitudinal dataset, the suggested E2CNN achieves accuracy rates of 98.31% ± 0.5% for non-disabled participants and 97.97% ± 1.41% for amputee subjects when applied to LMS-based images. The E2CNN outperforms the baseline CNN model by a significant margin of 24.67% on the NinaPro DB1 dataset, with an average accuracy of 91.27%. Compared to other CNN models and existing methods like stacked sparse autoencoders (SSAEs), the results show that the E2CNN approach is competitive. Because of its short training and prediction times, the E2CNN shows much promise for real-time sEMG classification using Log-Mel spectrogram pictures. To meet the needs of upper limb prosthesis applications in real time, the study provides an efficient and reliable neural network architecture for sEMG signal categorization.

The researchers combined feature vectors based on time, frequency, time–frequency (TF) characteristics, energy, and entropy^28^. CNN’s deep learning features from MFCC images were integrated with these for PCG classification. The authors claim that the changes in PCG signals caused by HVDs from specific angles can only be reflected in real-world applications by using features that have been carefully developed. More complete disease data may be collected when deep learning features with strong representation capabilities are merged. Intending to extract more discriminative features with fewer parameters, the authors of^28^ created a novel 2D CNN architecture for heart sound classification. The building in question utilized both channel-based and spatial forms of attention. PCG signals, spectrograms, and deep learning methods have all been used in recent research as well^14,15^.

**Table 1:** Analysis of prior work’s efficacy in classifying heart diseases.

Ref.	Features	Dataset	Models	Metrics
^16^	Spectrogram + CWT	PRV	RNN-LSTM	ACCURACY IS: 93%
^17^	Frequency based features	Physio-Net dataset	KNN-RF	ACCURACY IS: 95%
^18^	heart sounds segmentation features	Publically available Heart sound dataset	Euclidean distance (ED) and the its principles	ACCURACY IS: 96%
^19^	MFCC and DWT features	Pascal Challenge dataset	XGB, MFO and RF	ACCURACY IS: 89%
^12^	Multi-dimensional Scattering-transform	PRV	PCA-SVM	ACCURACY IS: 98%
^29^	MFCC	Physio-Net	ANN and LSTM	ROC_AUC IS: 91%
^30^	PCA feature selection	-	NN with PSO	ROC_AUC IS: 98%
^31^	EMD-PWPT features	PRV	RF	ACCURACY IS: 99%
^13^	Power Spectrum features	Phyio-Net	CNN	ACCURACY IS: 98.89%
^22^	Spectral-Statistical Features	NIH	NB, RF, SVM and kNN	ACCURACY IS: 97%

## Proposed approach

**Figure 1:**
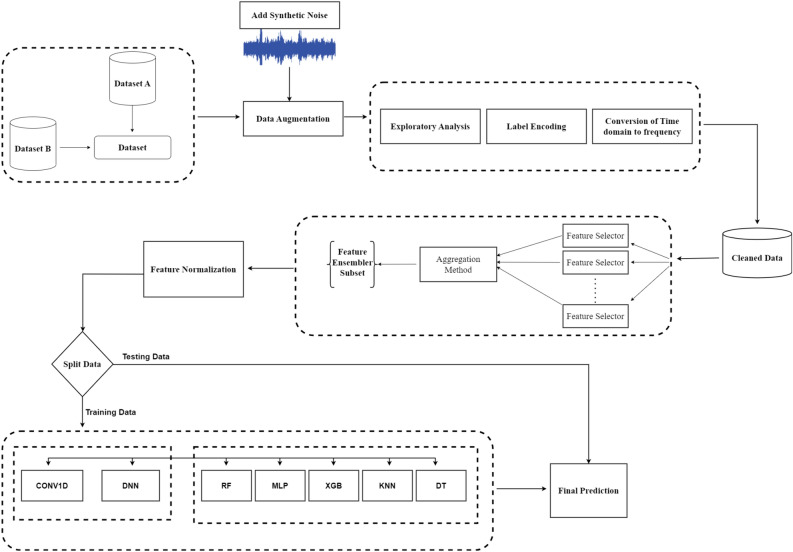
Proposed approach for AEDDB creation and abnormal event detection.

This study presents a novel approach for detecting heart disease using audio signals to optimize the detection technique. The schematic in Fig. 1 illustrates the proposed technique in a block diagram format. The proposed methodology encompasses a series of sequential stages: data acquisition, data augmentation, data pre-processing, feature extraction, feature normalization, model selection, model implementation, and result prediction. This study aims to enhance the reliability of the comparative analyses conducted in previous studies^8,9^. Consistency in the experimental setup and data collection methods is maintained throughout this investigation. The feature extraction process involves MFCCs, and eight other main feature extraction methods are employed to extract the most significant attributes from the dataset. Our methodology utilized a combination of ML and DL models to tackle the multi-classification problem in detecting heart disease. This investigation initially utilized the PASCAL classifying heart sound challenge Dataset and the 2016 PhysioNet/Computing in Cardiology (CinC) Challenge datasets. Ventricular Septal Defect (VSD), Atrial Septal Defect (ASD), Patent Ductus Arteriosus (PDA), murmur, and extrasystole are just a few of the disorders covered by the databases. The proposed methodology exhibits a heightened capacity for expedited disease detection and precision compared to previous research. The proposed methodology incorporates a total of five machine learning models, namely Random Forest (RF), K-Nearest Neighbour (KNN), Decision Tree (DT), Extreme Gradient Boosting (XGB), Multilayer Perceptron (MLP), and two deep learning models, Deep Neural Network (DNN), and 1D-Convolutional Neural Network (CNN1D). The evaluation of the model involved the consideration of various metrics, including accuracy, precision, recall, and the F1-score. Additionally, a confusion matrix was generated to comprehensively analyze the model’s performance concerning established benchmarks within the industry.

### Dataset selection and noise induction

The design project selected for this endeavor is the Classifying Heart Sounds Pascal Challenge (CHSPC). The dataset comprises a collection of heart sound recordings obtained from a sample size of 400 individuals. The participants were categorized into two groups: a group of 200 individuals with typical cardiac function and another group of 200 individuals with atypical cardiac function. The patients in the dataset were collected from four different clinical sites, each contributing an almost equal number of subjects. According to^32^, the dataset encompasses a maximum of three recordings, each lasting approximately 10 seconds, for every subject. These recordings are obtained from distinct chest positions. The WAV files contain audio recordings that were made with an electronic stethoscope. In addition to the recordings, the dataset also includes a set of annotations for each one, pinpointing where the heart sounds can be heard in the recording and classifying them as normal or abnormal. Professional cardiologists with years of experience annotated the data. Machine learning competitions used the CHSPC dataset to classify heart sounds as normal or pathological. This work aimed to create algorithms with the intelligence to analyze and classify heart sound recordings independently. The CHSPC dataset is a great resource for researchers and machine learning practitioners when building algorithms for identifying cardiac diseases using heart sound recordings.

**Figure 2:**
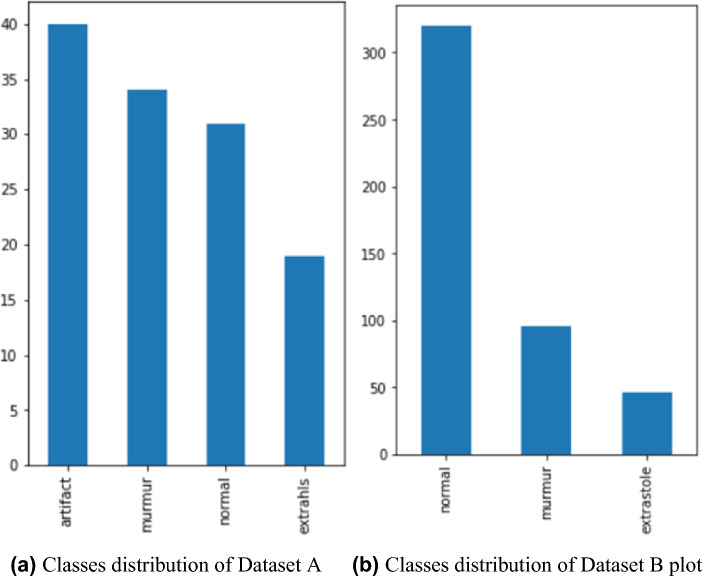
Classes distribution of datasets.

The contest consisted of two rounds. The proficiency of the participants’ segmentation algorithm abilities was assessed during the initial round. In contrast, the subsequent round focused on evaluating the algorithm’s effectiveness in accurately categorizing heart sounds as “normal,” “murmur,” “extra heart sound,” or “artifact” within a laboratory setting. To assess the efficacy of the novel methodology, only the outcomes derived from the initial segment of the experiment, encompassing both datasets, were considered for analysis. The algorithm’s robustness was tested using two datasets, including clean and noisy cardiac sounds. There are more audible heart sounds in the Digiscope data collection. The initial dataset consists of 175 audio signals, each belonging to one of four categories: “normal,” “murmur,” “extra heart sound,” or “artifact.” The distribution of classes in Dataset A is depicted in Fig. 2a. Dataset B comprises a total of 655 audio signals on heart disease. Dataset B consists of three distinct categories of audio signals, namely “normal,” “murmur,” and “extra stole.” The distribution of classes in Dataset B is depicted in Fig. 2b.

**Figure 3:**
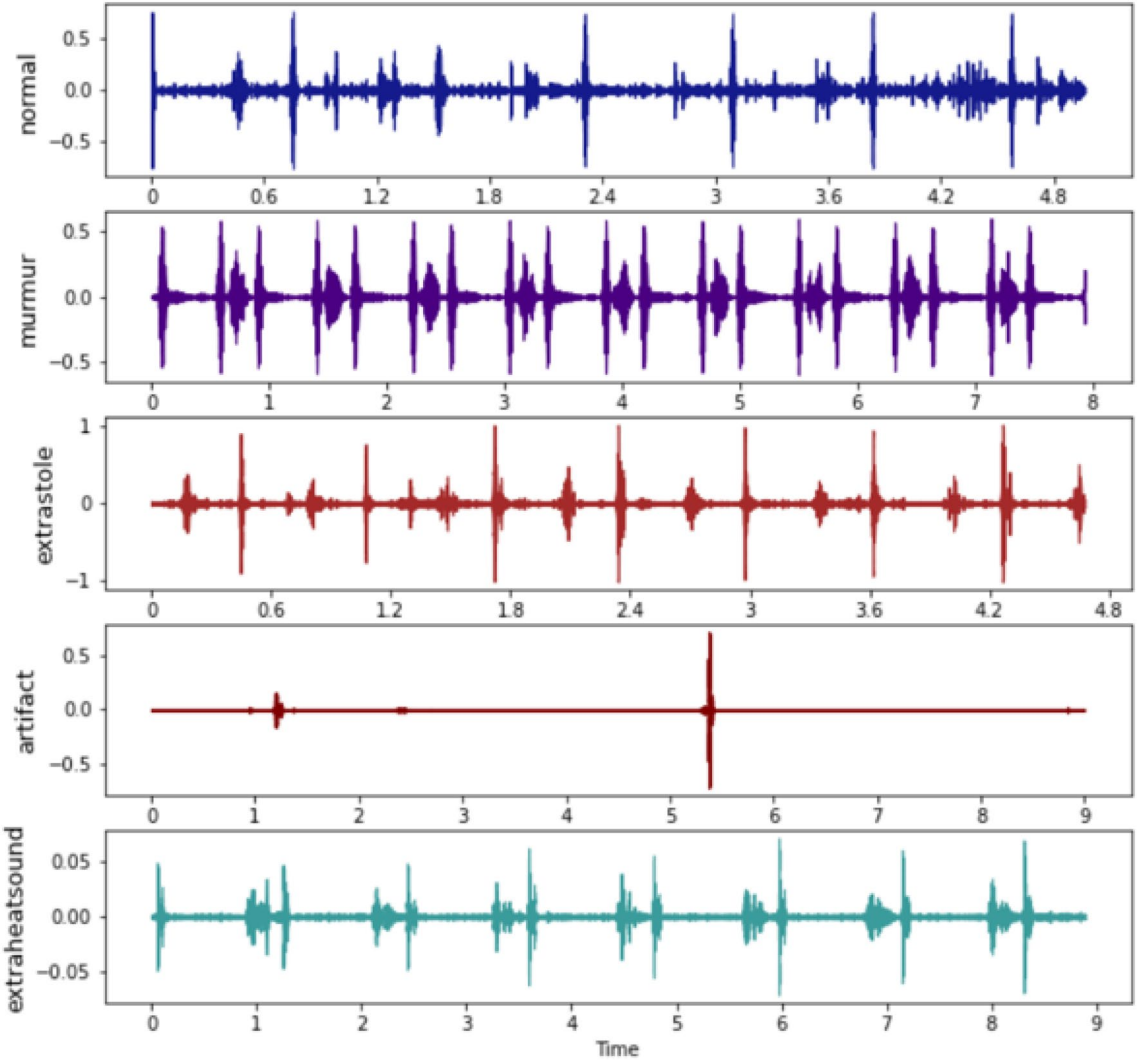
Waveforms of dataset audios.

The integration of both datasets was undertaken to enhance the complexity of this approach. The ultimate dataset comprises a total of 832 audio signals. Figure 3 illustrates the visual representation of the audio signals. The description of diseases presented in the dataset is shown in Table 2.

**Table 2:** Description of diseases present in dataset.

Heart Disease	Description
Normal audio signal	According to Liu et al.^32^, sounds produced by a healthy heart are classified as “Normal” under typical conditions. Upon removal of the recording device from the body, transient background noise may occur in the resulting audio recordings. There is potential for the presence of a diverse array of ambient sounds. In addition, it is possible for various incidental sounds, such as breathing or the inadvertent contact of clothing or body with the microphone, to be detected. A regular heartbeat exhibits a discernible rhythmic pattern characterized by the alternating sounds of “lub” and “dub”. Notably, the duration between successive “lub” sounds is comparatively shorter than between successive “dub” sounds, provided an individual’s heart rate remains below 140 beats per minute.
Murmur audio signal	heart murmur can be auscultated during either the systole or diastole phases of the cardiac cycle and are frequently described as resembling auditory sensations such as “whooshing, screaming, thundering, or turbulent fluid.” Several significant cardiac conditions exhibit this particular symptom during the interval between the first and second heart sounds, commonly called “lub” and “dub”. This symptom persists if left untreated. Individuals lacking medical training may experience confusion because murmurs manifest not during the occurrence of the first or second heart sound but rather in the temporal interval separating them.
Extrasystole	The auditory manifestation of extrasystole, wherein an additional or skipped cardiac contraction occurs, can sporadically be perceived and distinguished as a “lub-lub dub” or a “lub dub-dub” sound. (This phenomenon is distinct from an intermittent extra heartbeat.) Although extrasystoles can sometimes occur without indicating illness in adults and young children, they can also be associated with heart conditions and pose potential risks for adults. Therefore, it is important to investigate extrasystoles to thoroughly facilitate early diagnosis and treatment.
Artifact	According to Kumar and Saha^33^, a diverse range of sounds can be produced by artifacts, encompassing return squeals, echoes, speech, music, and noise. At frequencies lower than 195 hertz, the heart does not produce sounds that can be perceived by the human ear, resulting in minimal temporal regularity. In contrast to the other groups, artifacts exhibit the highest level of distinctiveness. The ability to differentiate this particular group from the preceding three is of utmost importance to facilitate potential re-evaluation or replication of the process.
Extra Heart Sounds	Additional cardiac sounds may manifest due to various cardiac pathologies, encompassing abnormalities affecting the heart’s valves, septum, or chambers. Upon observation, various interventions such as medications, lifestyle modifications, or surgical procedures may address additional heart sounds; however, the most appropriate course of action will be contingent upon the underlying cause. The effective management of this illness may necessitate heart monitoring and ongoing medical attention.

The utilization of Dataset A and Dataset B allows for establishing a uniform benchmark, facilitating the comparison of various algorithms and enabling researchers to replicate and further develop prior research endeavors. Moreover, including diverse pathological heart sounds within these datasets renders them highly valuable in developing diagnostic tools for cardiovascular conditions. The primary objective of this study was to investigate the detection of heart disease through the analysis of sound signals contaminated with noise. The study employed an existing publicly accessible dataset and generated a novel dataset by merging original and noisy heart disease sound signals. This new dataset facilitates further investigation and allows researchers to derive more significant findings from the data.

#### Noise induction and audio data augmentation

This research employed a data augmentation technique to enhance the generality and complexity of the dataset. Audio data augmentation refers to transforming current audio data into different variations. This technique enhances the generalization capabilities of machine learning models by exposing them to a diverse range of input data, thereby expanding the size of the training dataset. Many different modifications can be applied to audio data through audio augmentation techniques, such as changing the pitch or tempo, adding noise or other sound effects, adjusting the volume or balance, and performing time-stretching or time-shifting procedures. These techniques are flexible enough for audio information, such as music, sound effects, and speech. Audio data augmentation might be especially useful in applications where machine learning models’ high accuracy and robustness require extensive and diverse training datasets. Such uses can be seen in voice recognition, speaker verification, and music classification systems.

**Figure 4:**
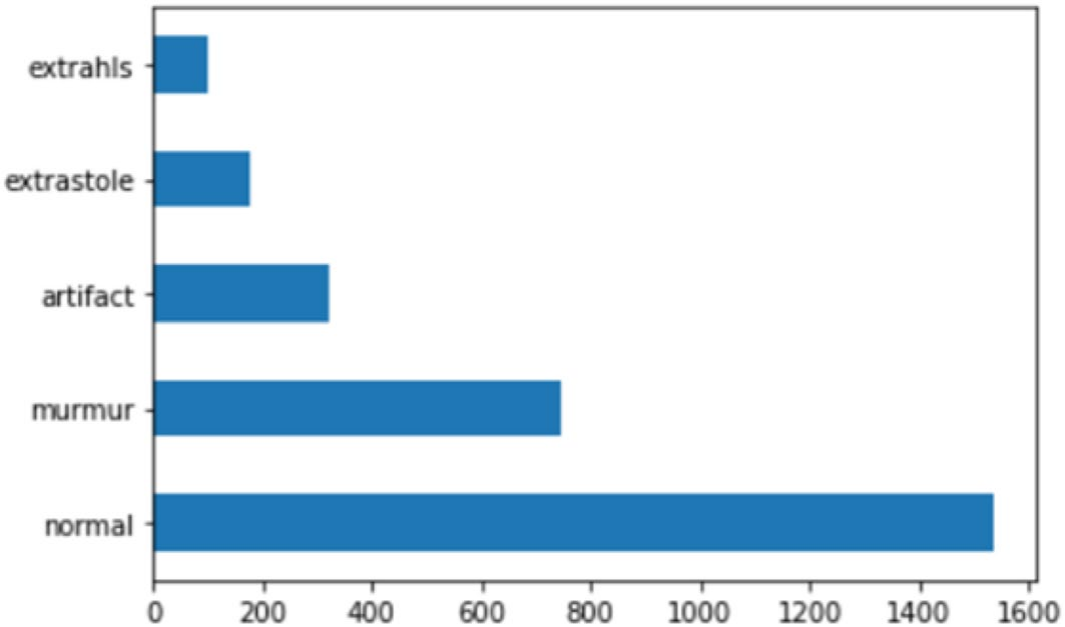
Final dataset classes distribution.

There were 832 different audio samples in the original dataset. Audio pitch and tempo changes and the addition of noise were among the methods used to supplement the data. The updated dataset now includes a total of 2882 audio sound signals, comprising 1538 “normal” signals, 746 “murmur” signals, 320 “artifact” signals, 176 “extrasystole” signals, and 102 “extra heart sounds.” Figure 4 shows the distribution of the final dataset used in the analysis.

### Data pre-processing

Pre-processing is a crucial step in ensuring optimal machine learning model performance. The audio data underwent several preprocessing stages before integrating into the training phase. The initial stage in editing audio data involves converting it into a format that can be understood by a computer, thereby enabling the extraction of essential values in subsequent steps.

#### Sampling rate

A sample refers to a discrete subset of data, exemplified by a fragment of audio lasting briefly, typically measured in seconds. The sample rate describes the frequency at which samples are collected. The sample rate (frame rate) utilized in our study was 44100. The equation 1 allows the total number of frames in an audio file to be calculated by multiplying the sampling (frame) rate by the file’s duration in seconds^9^.

1 $$\begin{aligned} \,total\,frame = samplingrate \times time \end{aligned}$$

If the signal labeled as “file1” is an analog signal that spans 9 seconds, we can utilize Equation 2 to calculate its overall frame rate.

2 $$\begin{aligned} file1 = 44100 \times 9 \end{aligned}$$

#### Data framing

**Figure 5:**
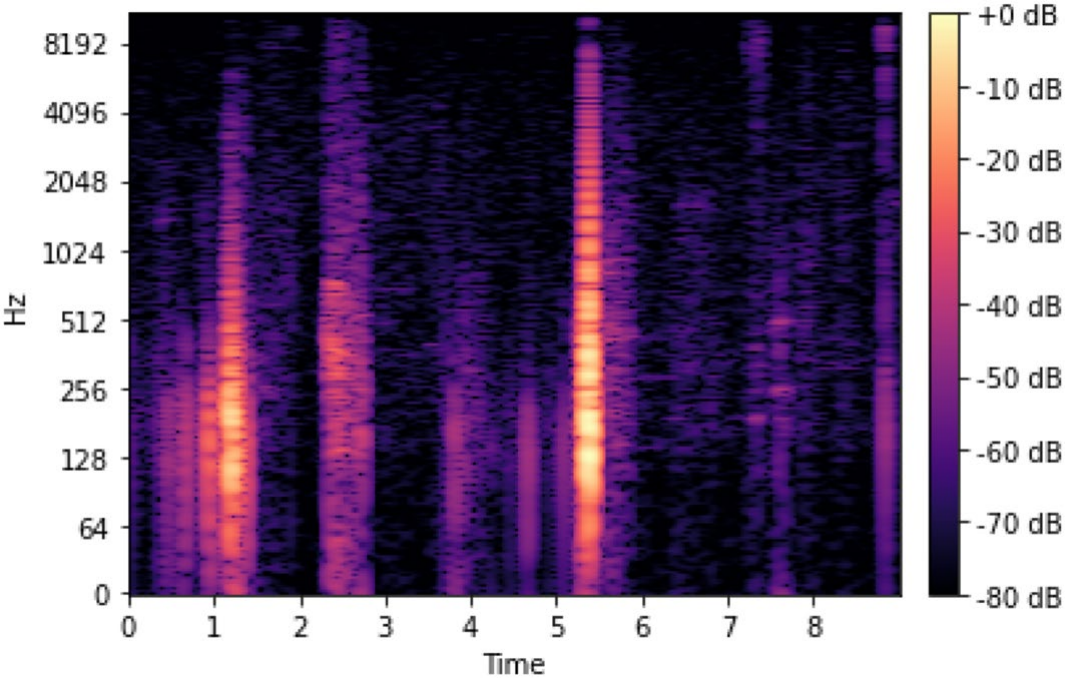
Conversion of the time domain to frequency.

Data framing is a technique that can be employed to ensure uniformity in the sampling (frame) rate of all audio files. The initial stage in sound processing often involves extracting pertinent acoustical features, followed by decision-making processes encompassing information acquisition, categorization, and integration. Subsequently, the data derived from the audio signal is converted into a format appropriate for depiction in an alternative domain, namely the frequency domain. It was determined that a greater sampling rate and a significantly larger number of data points were required to depict audio data effectively. Samples indicate the magnitude of an audio waveform at a particular moment in time. Figure 5 displays a mel-spectrogram of a synthetic audio file, showing how the “loudness” of the signal changes over time at various frequencies. The horizontal axis of the audio clip represents time, specifically 9 seconds. On the other hand, the vertical axis displays frequencies ranging from 0 to 8 kHz. The mel-spectrogram visually represents a sound wave’s amplitude by using purple hues.

#### Data normalization and encoding

Data normalization refers to scaling numerical data to a standardized scale or range. This practice is to mitigate the influence of variations in scale on the analysis and processing methods employed for data. Normalization techniques are utilized in machine learning, statistics, and data mining. The data normalization in this study was conducted using the standard scalar method. The utilization of the standard scalar normalization technique is prevalent in the field of machine learning. The pre-processing stage involves standardizing the characteristics by subtracting the mean and dividing by the standard deviation. The resulting data collection is transformed such that each characteristic has been adjusted to have a mean of zero and a standard deviation of one. The utilization of Standard Scalar is particularly advantageous in cases where the dimensions of the features within the dataset exhibit variations, as this discrepancy can potentially impair the efficacy of numerous machine learning methodologies. When utilizing the Standard Scalar, the features will possess a uniform scale, facilitating their comparability and evaluability. The present study employs an Equation 3 to standardize the feature set of the dataset.

3 $$\begin{aligned} { X^{'} = \frac{(X - X\_mean)}{X\_std}} \end{aligned}$$

Let *X* represent the original feature, $X\_mean$ denote its mean, $X\_std$ represent its standard deviation, and $X^{'}$ denote its standardized version. In addition, the process of converting categorical variables into numerical representations is accomplished by utilizing the One-Hot-Encoder feature transformation technique.

### Feature extraction

**Figure 6:**
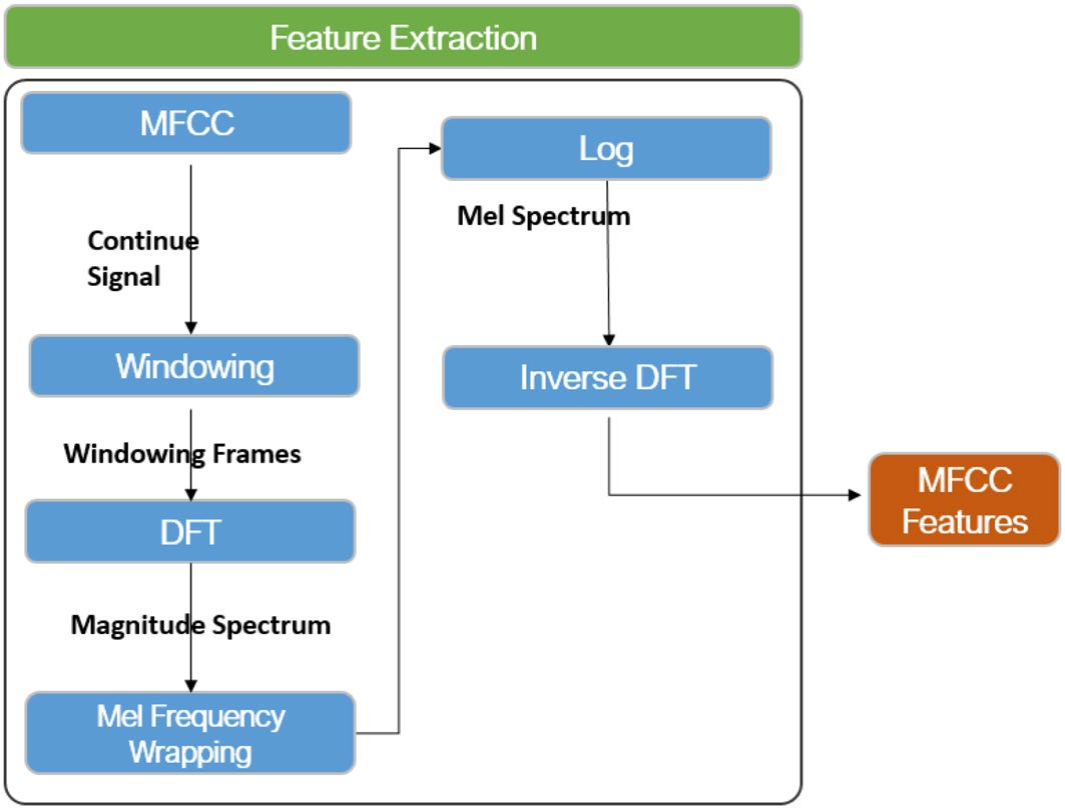
MFCC feature extraction process.

Multiple characteristics can be discerned within each sound wave. However, we must emphasize the specific aspects of the forthcoming event we intend to unveil. The initial stage of this analysis involved the utilization of Mel Frequency Cepstral Coefficients (MFCC). The process of extracting MFCC features is depicted in Fig. 6, with each step being elucidated subsequently. The process of extracting Mel-frequency MFCC features is succinctly outlined in this section.

*Audio Preparation* step involves applying preprocessing techniques to the audio stream to eliminate background noise and non-speech or silent intervals.

*Framing* is a step that occurs after preprocessing, where the signal is divided into shorter frames that typically last between 20 to 30 milliseconds. There is usually some overlap between consecutive frames. The temporal variations of the signal can be captured, leading to an enhancement in temporal resolution.

*Windowing* is a technique to mitigate spectral leakage and emphasize essential information within each frame. This is achieved by applying a window function, such as the Hamming window, to the frame. The choice of window size is 25 milliseconds to make short segments of the audio signal before computing the MFCCs. A common choice is 25 milliseconds, equivalent to 400 samples.

*Fourier transform*, specifically the short-time Fourier transform (STFT), is utilized to convert the windowed frames into the frequency domain. This transformation results in a set of spectra with complex values. For a window length of 25 milliseconds, corresponding to 400 samples, we use 512 1024 FFT points.

*Mel-frequency wrapping* is a technique developed to approximate the non-linear frequency response of the human ear. This technique utilizes a perceptual frequency scale known as the Mel scale. A filter bank consisting of triangle filters is employed to map the amplitude of each spectrum onto the Mel scale. These filters are designed with narrower spacing at lower frequencies and wider spacing at higher frequencies.

*Logarithmic compression* is employed to compress the dynamic range and accentuate the distinctions among the filter-bank coefficients by taking the logarithm of the magnitude values in each Mel filter-bank.

*The Discrete Cosine Transform (DCT)* is employed to convert the coefficients of the resulting log-Mel filter bank to the cepstral domain, enabling their utilization. Typically, only the coefficients with the lowest order are preserved as they effectively capture the fundamental characteristics of the signal.

*The delta and delta-delta* features can be obtained by computing the first and second derivatives of the MFCCs. The aforementioned characteristics capture the temporal progression of the MFCCs and have the potential to provide further insights into the dynamics of the signal.

In addition to employing the MFCC feature extraction method, this study incorporated nine additional feature extraction methods and developed a feature ensembler. This study applies various feature extraction techniques: spectral centroid, spectrum, zero-cross examination rate, spectral bandwidth and spectrum roll-off. These methods are presented in Table 3.

**Table 3:** Feature Matrix using various methods.

Feature Matrix	Methods	Mean (trunc)	Std (trunc)	Median (trunc)	Skew (trunc)
F_0	MFCC 0 - 39	$\checkmark$	$\checkmark$	$\checkmark$	$\checkmark$
F_1	zero_crossing_rate	$\checkmark$	$\checkmark$	$\checkmark$	$\checkmark$
F_2	spectral_rolloff	$\checkmark$	$\checkmark$	$\checkmark$	$\checkmark$
F_3	spectral_centroid	$\checkmark$	$\checkmark$	$\checkmark$	$\checkmark$
F_4	spectral_contrast	$\checkmark$	$\checkmark$	$\checkmark$	$\checkmark$
F_5	spectral_bandwidth	$\checkmark$	$\checkmark$	$\checkmark$	$\checkmark$
F_6	chroma_stft	$\checkmark$	$\checkmark$	$\checkmark$	$\checkmark$
F_7	RMS	$\checkmark$	$\checkmark$	$\checkmark$	$\checkmark$
F_8	mel-spectrogram	$\checkmark$	$\checkmark$	$\checkmark$	$\checkmark$

*Zero Crossing Rate:* The pace at which the sign of a signal change can be used as an indicator of how noisy or clean the sound is; this rate is known as the zero crossing rate. More high-frequency content or noise is associated with a higher zero crossing rate, while a smoother and less noisy signal is associated with a lower rate^34^.

*Spectral Roll-off:* It is the frequency at which a given fraction of the total spectral energy is located. It aids in defining the spectral profile of a sound. Most of the energy is concentrated at low frequencies if the spectral roll-off is small, while a larger number implies a more even distribution over the audible spectrum^35^.

*Spectral Centroid:* The “brightness” of an audio signal can be determined by calculating the spectral centroid, which is the spectrum’s mathematical center of mass. A high spectral centroid value indicates that the audio is treble- or high-heavy, whereas a low value indicates that the music is bass- or low-heavy^36^.

*Spectral Contrast:* The difference in amplitude between peaks and valleys in the spectrum is measured by spectral contrast. It is used to determine the prominence of various spectral peaks. A higher contrast value suggests sharper spectral peaks, implying different sound components^36^.

*Spectral Bandwidth:* The spectral bandwidth measures the spectral content’s width and the audio signal’s frequency spread. While a low value indicates a lower concentration of frequencies, a high value denotes a wide distribution of frequencies^37^.

*Chroma STFT:* The harmonic content of the audio is represented by the chroma Short-Time Fourier Transform (STFT). The study of tonal qualities and musical notes is made possible by the extraction of information about the pitch class of audio frames^37^.

* Root Mean Square (RMS):* measures an audio signal’s root mean square amplitude. It divulges details about the signal’s overall energy level. A louder audio is indicated by a greater RMS value^38^.

*Mel-Spectrogram:* is a representation of the audio signal in the mel-frequency domain is a mel-spectrogram. It facilitates human-like audio analysis by converting the linear frequency scale into a perceptually appropriate mel-frequency scale^34^.

To determine the value of each element, a computation is performed by taking the average of all the numbers obtained within each frame, followed by the subtraction of their respective standard deviations. A spectrum energy map was generated for the Mel scale by applying the Fourier transform (using the window abbreviation) to the signal. This was done using the MFCC series of infant audio recordings. Subsequently, by employing an independent cosine transformation on the Mel log energy array, extract the logarithms of the power values. MFCCs represent the intensities of the emerging spectrum. The present study introduces a novel feature ensembler. The ensembler in question incorporates a collection of features derived from various feature extraction methods. The data frame contains 288 elements extracted from each audio file. In our approach, we first used the Standard Scaler, a standard normalization technique, to normalize the features in our dataset. Normalization is essential for ensuring that the various features are on a similar scale and that one does not dominate the others during the modeling process. This step enhances the model’s stability and performance. Following normalization, we transformed the features into a numpy array, a data structure ideal for numerical computations and analysis. When working with data, Numpy arrays provide efficiency and flexibility. Following that, we reshaped the data to meet the needs of our chosen machine-learning models. Data shaping ensures compatibility and consistency when sending data to models.

Finally, we separated our data into training and testing sets. The training set is used to train our classification models so that they can learn and predict. The test set, which the models did not see during training, assesses their performance and generalization to new, previously unseen data. This methodical approach, from normalization to data conversion, reshaping, and splitting, lays the groundwork for successful classification model application to our dataset. It ensures that the models are appropriately trained and rigorously evaluated for their classification tasks, contributing to our results’ overall quality and reliability.

### Classification models and parameters settings

The study employed five machine learning models, specifically Random Forest (RF), K-Nearest Neighbour (KNN), Decision Tree (DT), Extreme Gradient Boosting (XGB), and Multilayer Perceptron (MLP). Two deep learning models, Deep Neural Network (DNN) and 1D-Convolutional Neural Network (CONV-1D), were also utilized. Each model is described individually, providing detailed information about its fine-tuned parametrized settings.

#### Machine learning models

This section provides the machine learning models used for experiments.

*Random Forest Model:* is an ensemble learning model commonly employed for various tasks, including classification and regression. The ensemble model consists of a collection of decision trees constructed using a randomly selected set of features and training data. The mentioned characteristics of the subject are widely recognized in academic circles, including its exceptional precision, robustness against distortions and anomalies, and ability to handle data with a high number of dimensions effectively. This method has been successfully implemented in various industries, such as banking, medicine, and bioinformatics. The methodology employed involves the generation of multiple decision trees during the training process, followed by determining the class that represents the average of all the predicted classes, specifically in the context of regression. The estimation of the significance of features is also possible. The experimental settings for the RF model include the following parameters: maximum depth of 8, the maximum number of features considered for splitting at each node set to 5, the minimum number of samples required to split an internal node set to 5, and the number of estimators (i.e., decision trees) in the random forest ensemble set to 500.

*Decision Tree Model*: is a supervised learning algorithm commonly employed in machine learning to classify problems. The system’s functioning involves dividing the dataset into smaller subsets, utilizing a predetermined set of features. This partitioning is performed recursively, further dividing the subsets into even smaller subsets until the data can be readily classified. The construction of the tree structure involves the iterative partitioning of the dataset into increasingly smaller subsets, guided by the optimal feature values that effectively distinguish between different classes. The outcome is a tree structure consisting of decision nodes and leaf nodes. The decision nodes hold the split conditions, while the leaf nodes store the class labels. Decision trees have gained popularity due to their ease of comprehension, interpretability, and ability to accommodate both categorical and numerical data. Nevertheless, it is worth noting that these models tend to fit the training data excessively, thereby compromising their generalization capabilities. Consequently, ensemble methods such as random forests are frequently employed to enhance their overall performance. The parameters of the DT model were set as criterion=’gini’, splitter=’best’, max_depth=None, min_samples_split=2, min_samples_leaf=1, and min_weight_fraction_leaf=0.0 throughout the experiments.

*Extreme Gradient Boosting Model:* XGBoost, a widely adopted gradient boosting technique, holds prominence in machine learning for its application in classification and regression tasks. The XGBoost algorithm is based on the gradient boosting framework, which involves iteratively adding models to an ensemble. Each added model aims to improve the overall performance of the ensemble by reducing the errors made by the previous models. XGBoost differs from previous gradient boosting techniques by incorporating the ability to handle missing values in the input data and employing a more regularized model formulation to mitigate the issue of overfitting. The acceleration of model training is achieved by using parallel processing techniques and implementing a more efficient optimization approach. Due to its inherent attributes, XGBoost has gained significant popularity and proven to be a highly efficient machine learning technique, particularly suitable for tasks involving the analysis of extensive datasets and complex feature spaces. The default parameters of the XGB model were maintained throughout the experiments.

*Multilayer Perceptron Model: * consists of several layers of interconnected nodes or neurons. These layers include an input layer, several hidden layers, and an output layer. Neurons in the layer above them feed the neurons in network information. After processing them, a non-linear activation function is applied to these signals by producing a weighted total. Finally, the processed signals are transmitted to the next network layer. To accomplish this, the inter-neuron connection weights are frequently acquired through a backpropagation technique. Supervised learning encompasses various problem domains, two of which are classification and regression. MLPs have demonstrated exceptional performance in these specific domains. The parameters of the MLP model utilized in the experiments were maintained at their default values.

*K-Nearest Neighbours Model:* is a fundamental machine learning algorithm employed in classification and regression tasks. The algorithm identifies the K nearest labeled data points to a novel, unlabeled data point and leverages their class or average value to generate predictions. The algorithm operates under the assumption that data points that exhibit similarity are likely to possess similar labels or values. The selection of the parameter K influences the adaptability of the decision boundary. The K-NN algorithm is characterized by its simplicity in comprehension and implementation. However, it is important to note that its computational demands can increase significantly when applied to large datasets. Additionally, the performance of K-NN is susceptible to the scaling of features. The algorithm generally exhibits versatility by effectively capturing local patterns within the dataset. The parameters of the KNN were set as n_neighbors=5, weights=’uniform’, algorithm=’auto’, leaf_size=30, p=2, metric=’minkowski’, metric_params=None and n_jobs=None.

#### Deep learning models

In recent years, the widespread integration of deep learning and machine learning models has transformed many fields, providing unprecedented answers to complex challenges. Convolutional Neural Networks (CNNs) and other deep learning architectures are used in fields ranging from computer vision and natural language processing to medical diagnostics and financial predictions. Researchers have used the power of these sophisticated models to extract subtle patterns and representations from big information, allowing breakthroughs in a wide range of applications^39,40^. As the demand for intelligent systems grows, the exploration and implementation of these models remain at the leading edge of cutting-edge research and innovation. This research dives into using advanced deep learning techniques, focusing on CNNs, to address a paradigm that highlights the versatility and efficacy of modern neural network architectures.

The two significant deep learning models utilized in this study are the DNN and the CONV-1D. Each model is described separately, providing detailed information about their fine-tuned parametrized settings.

*One-dimensional Convolutional Neural Network:* The present study utilized 1D-CNN. The Conv1d architecture is widely utilized in deep learning for processing sequence data with a singular dimension. This encompasses various data types, such as time series, audio signals, and textual information. In a Conv1d network, individual convolutional layers acquire distinct filters that are subsequently closely integrated with the input signal to discern patterns or features. Rectified Linear Unit (ReLU) is one example of a non-linear activation function used at the end of each convolutional layer to give the model its non-linearity. The convolutional layers are typically followed by one or more fully connected layers responsible for performing the classification or regression task. The Conv1d network employs the backpropagation optimization process to train its parameters. This process involves adjusting the network’s weights and biases to minimize a loss function, quantifying the discrepancy between the anticipated and observed output.

The CNN model under examination comprises three convolutional layers, three max-pooling layers, two dropout levels, and two fully connected layers. Dropout is a regularization technique used in deep learning to address the problem of overfitting. Overfitting occurs when a model becomes excessively complex during training on a limited dataset, fitting the noise rather than the underlying pattern. As a result, extrapolating to unfamiliar data becomes challenging. The model underwent training using 571,525 parameters. In the context of training neural networks, deep learning commonly utilizes optimizers and employs the categorical cross-entropy loss function. Optimizers iteratively adjust the parameters by changing the weights and biases of a neural network during training. Optimization aims to find the values of the weights and biases that provide the least deviation between the predicted and actual output (the loss function). Among the many optimization methods available are stochastic gradient descent, Adam, RMSProp, and many more. In multiclass classification applications, categorical cross-entropy is a popular loss function. The statistical metric quantifies the discrepancy between the actual probability distribution for a particular class and the estimated distribution. The primary goal is to optimize the probability of correctly classifying an instance while minimizing the categorical cross-entropy loss. During the training process, the weights and biases of a neural network are iteratively updated by an optimizer in conjunction with a loss function, such as categorical cross-entropy. This iterative update process aims to enhance the network’s predictive capabilities. The present study employed the Adam optimizer and utilized categorical cross-entropy as the loss function during the model’s training. The experimental configuration involved setting the batch size to 64 and the number of epochs to 90.

*Deep Neural Network:* Artificial neural networks, specifically Deep Neural Networks (DNNs), are widely utilized due to their extensive layers of computational capacity. Each layer within the network is designed to acquire a progressively intricate representation of the data by building upon the preceding layers. The term “input layer” denotes the layer in a neural network closest to the input data, while the term “output layer” denotes the layer closest to the output. The term “hidden layers” pertains to the intermediate layers between the observable layers. DNNs have demonstrated their ability to effectively address complex problems such as image classification, natural language processing (NLP), and speech recognition. The individuals undergo training using large datasets, employing algorithms that modify the weights and biases of the model and are subsequently assessed using a loss function. The architecture incorporated seven dense layers. No dropout layers were employed to compare a generalized model with a complex one. All other experimental conditions remain consistent with those of the CNN model.

The DNN model is structured sequentially, showing a typical classification task architecture with tightly connected layers. Following an initial layer with input data-aligned dimensionality, successive layers gradually decrease the number of neurons. To add non-linearity, the ReLU activation function is used by 1000 neurons in the first dense layer. This layer inputs weights and biases totaling 289,000. In the same way, the following dense layers consist of 750, 500, 250, 100, 50, and 5 neurons, respectively. The output layer for multiclass classification is the last dense layer with 5 neurons.

There are a total of 1,570,905 trainable parameters in the architecture. These parameters are fine-tuned throughout training to maximize the model’s capacity to identify links and patterns in the input data. The model can capture complex patterns in the dataset since the number of neurons in each layer decreases, making it easier to extract hierarchical features. Although the output layer’s activation function is not specified, multiclass classification tasks frequently employ softmax to generate probability distributions for each class. This DNN model is well-suited for classification tasks since it strikes a good mix between being overly complicated and being able to detect complex patterns in the input data.

## Experimental results and discussion

This section presents a comprehensive account of the research findings and outlines the experimental setup employed in the study. This proposed method aims to assess the presence of four distinct types of heart disease, namely “normal,” “murmur,” “extrasystole,” “extra heart sound,” or “artifact” within an audio dataset. This study proposes implementing a standardized methodology for identifying and isolating heart disease indicators within audio signals while effectively mitigating the influence of background noise. In our testing, we evaluated the performance of various machine and deep learning models. Five often used metrics are the confusion matrix, accuracy, precision, recall, and F1-score, all used to evaluate performance in testing situations. These stated performance criteria can be used to evaluate the machine learning model. The outcomes of the experiments are compared to those found using other approaches and in earlier studies.

### Experimental settings and evaluation metrics

The experimental parameters employed in this study are delineated herein. The work utilizes the Kaggle cloud-based platform, which offers complimentary access to GPU resources. Python is employed to conduct experiments. In our study, we have employed the Windows 10 operating system, which is equipped with a 2.30GHz Intel(R) Xeon(R) CPU. Kaggle is equipped with NVIDIA TESLA P100 Graphics Processing Units (GPUs), which exhibit notably superior processing speed compared to a typical personal computer’s Central Processing Unit (CPU). The experiments were conducted utilizing Python 3.8.8.

To assess the model’s performance on the test data, this research utilizes commonly accepted criteria for evaluating machine learning models. These indicators function as measurable metrics of the model’s effectiveness, facilitating the identification of problematic areas with greater ease. Evaluation metrics are utilized to assess and compare different models and optimize the hyper-parameters of a model to achieve the best possible performance. They assist in evaluating the strengths and weaknesses of the model, which can subsequently inform efforts to enhance it. By employing evaluation metrics, insights can be gained regarding the model’s capabilities and limitations, facilitating the implementation of refinements to enhance its efficacy in real-world scenarios. Accuracy, precision, recall, f1-score, and the confusion matrix are only a few of the metrics used in machine learning to assess the performance of a classification model. This study employed multiple evaluation metrics, as delineated in the following section.

*Accuracy* is a quantitative measure used to evaluate the overall performance of classifiers. It is calculated by determining the percentage of correct predictions concerning the number of instances. The following is the formula for determining accuracy in equation 4:

4 $$\begin{aligned} Accuracy = \frac{TP + TN}{TP + FP + TN + FN} \end{aligned}$$

In a multi-classification context, True Positive (TP) refers to a situation where a positive outcome is correctly identified, while True Negative (TN) denotes the correct identification of a negative outcome. Conversely, False Positive (FP) signifies an incorrect identification of a positive outcome, and False Negative (FN) represents an erroneous identification of a negative outcome. In the present context, the variables TP, TN, FP, and FN are employed to denote the quantities of accurate positive predictions, accurate negative predictions, inaccurate positive predictions, and inaccurate negative predictions made by the model.

*Precision *of a classifier is determined by the ratio of correct predictions to the total number of positive predictions made by the classifier. The precision rating can be determined using the equation 5 provided below:

5 $$\begin{aligned} Precision = \frac{TP}{TP + FP} \end{aligned}$$

*Recall*, alternatively referred to as sensitivity, quantifies the ratio of correctly identified positive predictions to the total number of positive instances. It can be computed using the subsequent mathematical expression shown in equation 6:

6 $$\begin{aligned} Recall = \frac{TP}{TP + FN} \end{aligned}$$

*F1-score* represents the optimal balance between accuracy and memory utilization. The optimal F1-score is 1, while the lowest attainable score is 0. The metric is computed using a weighted average of the precision and recall scores. Presented below in equation 7 is a mathematical expression that can be utilized to compute the F1-score.

7 $$\begin{aligned} F1\text {-}score = 2 * \frac{Precision * Recall}{Precision + Recall} \end{aligned}$$

*Confusion matrix:* is a tabular representation that provides a concise overview of the classification model’s performance, achieved by juxtaposing the observed and predicted values. The set of values consists of four components, namely true positives (TP), true negatives (TN), false positives (FP), and false negatives (FN). The matrix rows correspond to the true class labels, whereas the columns correspond to the predicted class labels. The primary diagonal of the matrix corresponds to the instances that have been accurately classified, whereas the elements outside the diagonal represent instances that have been classified incorrectly. These metrics serve the purpose of identifying the strengths and weaknesses of the model, thereby facilitating its improvement to attain superior outcomes.

### Data splitting criteria

When evaluating data mining techniques, it is imperative to establish distinct training and testing sets for the machine learning models. Verifying the model’s predictions becomes easier when the testing set contains data about the relevant attribute. The dataset will undergo an initial pre-processing phase before being divided into training and test sets. The data is subjected to analysis, and subsequently, the model undergoes a training process to enable it to generate predictions. The utilization of test data enables the evaluation of the performance of the training data. The complete dataset was divided into two distinct groups. The initial step involves partitioning the original training dataset into two equal parts. In the training phase, approximately 80% of the entire dataset was utilized. The second partition allocated a proportion of 20% from the entire dataset for testing.

### Experimental results with original dataset

Table 4 shows the experimental findings with the original dataset, as well as the performance metrics for various ML models. Among the models tested, RF had an accuracy of 88.58%, with precision, recall, and F1-score all at 89.58%. The equivalent *P*-value for RF is 1.0 decimals, showing no significant departure from baseline performance. Moving on to the MLP model, it achieved a superior accuracy of 94.53%, with consistent precision, recall, and F1-score metrics and a *P*-value of 1.0 decimals, indicating no significant divergence from the baseline.

XGBoost demonstrated outstanding performance with an accuracy of 94.11%, similar to the high precision, recall, and F1-score values, alongside a *P*-value of 1.0 decimals. K-Nearest Neighbors demonstrated balanced performance with an accuracy of 83.39% and a *P*-value of 0.71 decimals, indicating a statistically significant difference from the baseline. The DT model produced a great accuracy of 94.84%, which was supported by strong precision, recall, and F1-score metrics, as well as a statistically significant *P*-value of 0.99 decimals.

In the field of deep learning methodologies, the Conv1D model demonstrated excellent performance, obtaining an accuracy of 95.20%, as well as precision, recall, and F1-scores of 95.20%. Unfortunately, the *P*-value for Conv1D was not published. Finally, the DNN achieved an accuracy of 84.08% while adjusting the precision (89.10%), recall (84.08%), and F1-score (85.10%) measures.

**Table 4:** Experimental Results with Original Dataset.

Models	Accuracy (%)	Precision (%)	Recall (%)	F1-score (%)	*P*-value (decimals)
RF	88.58	89.58	89.58	89.58	1.0
MLP	94.53	94.53	94.53	94.53	1.0
XGB	94.11	94.11	94.11	94.11	1.0
KNN	83.39	83.39	83.39	83.39	0.71
DT	94.84	94.84	94.84	94.84	0.99
Deep Learning Approaches					
Conv1D	95.20	95.20	95.20	95.20	-
DNN	84.08	89.10	84.08	85.10	-

### Experimental results with augmented dataset

#### Machine learning approach results

**Figure 7:**
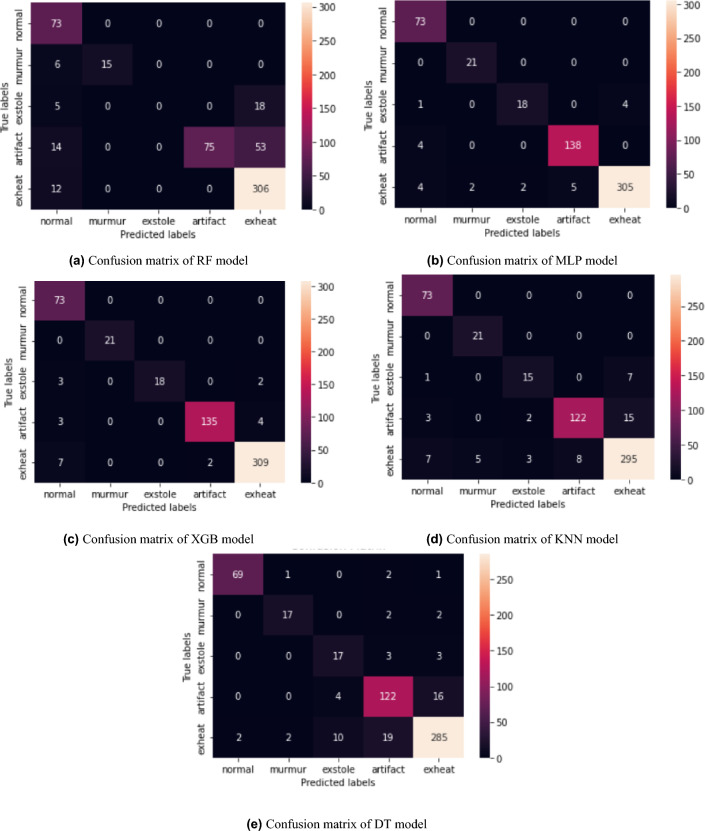
Confusion matrix of machine learning models.

This section explains the numerical outcomes of each machine learning model as presented in Table 5. The confusion matrix of machine learning models is depicted in Fig. 7.

Table 5 shows the results of the machine learning models. The RF model demonstrated an accuracy rate of 80.92%, precision rate of 81.90%, recall rate of 81.90%, and an F1-score of 81.89%. The *P*-value for the RF model is 0.67 decimals. The performance of the MLP model was notably superior, achieving an accuracy rate of 95.65%, *P*-value of 1.0 decimals, precision rate of 96.60%, recall rate of 97.60%, and an F1-score of 96.60%. Similarly, the XGB model exhibited robust performance, achieving an accuracy rate of 95.31%, a *P*-value of 1.0 decimals a precision rate of 96.30%, a recall rate of 97.31%, and an F1-score of 96.30%. The KNN model attained an accuracy rate of 91.16%, a precision rate of 91.15%, a recall rate of 91.15%, and an F1-score of 91.14%. The *P*-value for the KNN model is 0.85 decimals. The DT model achieved an accuracy rate of 88.38%, a precision rate of 88.37%, a recall rate of 88.37%, and an F1-score of 88.36%. Finally, the *P*-value for the DT model is 0.97 decimals.

**Table 5:** Machine learning numerical results with augmented dataset.

Models	Accuracy (%)	Precision (%)	Recall (%)	F1-score (%)	*P*-value (decimals)
RF	80.92	81.90	81.90	81.89	0.67
MLP	95.65	96.60	97.60	96.60	1.0
XGB	95.31	96.30	97.31	96.30	1.0
KNN	91.16	91.15	91.15	91.14	0.85
DT	88.38	88.37	88.37	88.36	0.97

The confusion matrix of the RF model in Fig. 7a presents a concise overview of its classification efficacy across various categories of heart disease. The confusion matrix offers a comprehensive analysis of the model’s predictions for each class, presenting the quantities of true positives, true negatives, false positives, and false negatives. Assessing the model’s performance involves evaluating its ability to accurately classify each class, identifying instances of misclassification, and gaining insights into the RF model’s capabilities and limitations in classifying various forms of heart disease. The diagonal elements of the confusion matrix represent the correctly classified instances, while all other elements indicate instances misclassified by the RF model. The XGB and MLP confusion matrix is depicted in Fig. 7b,c. The MLP and XGB models demonstrated superior performance across all performance metrics within the model set. The exhibited superior classification performance suggests their effectiveness in accurately identifying and differentiating the different forms of heart disease based on the audio signals. In contrast, the RF, KNN, and DT models exhibited comparatively diminished accuracy, precision, recall, and F1-score performance. Analyzing the confusion matrix of the KNN model in Fig. 7d aids in gaining insights into the strengths and weaknesses of the KNN model in accurately predicting heart disease based on the provided audio data. The confusion matrix comprehensively evaluates the model’s classification performance for each class. Although the KNN model demonstrated commendable accuracy in certain categories such as “normal” and “murmur,” it faced difficulties in accurately discerning between specific classes such as “extrasystole,” “artifact,” and “exheart.” The observed misclassifications suggest that there is room for improvement in the model’s performance in accurately categorizing various types of heart disease. This could be achieved through additional optimization or feature engineering techniques. The confusion matrix of the DT model in Fig. 7e offers valuable insights into its classification performance across various heart disease categories. The DT model demonstrated a satisfactory level of accuracy in the classification of instances labeled as “artifact” and “exheart”. Nevertheless, the system faced difficulties in accurately differentiating between the “normal,” “murmur,” and “extrasystole” categories, as evidenced by the misclassifications observed in the confusion matrix.

The notable efficacy of the MLP and XGB models can be ascribed to their aptitude for capturing intricate patterns and interconnections within the dataset. The MLP model utilizes a multilayer architecture to acquire complex representations, whereas the XGB model employs gradient-boosting techniques to improve its predictive abilities. The flexibility and adaptability exhibited by these models render them highly suitable for heart disease detection.

It is crucial to acknowledge that selecting the most appropriate model is contingent upon particular factors, including computational complexity, interpretability, and the specific context of the heart disease detection application. The process of assessing trade-offs and ensuring that the model’s characteristics align with the task’s requirements is crucial when choosing the most suitable model.

#### Deep learning approach results

**Figure 8:**
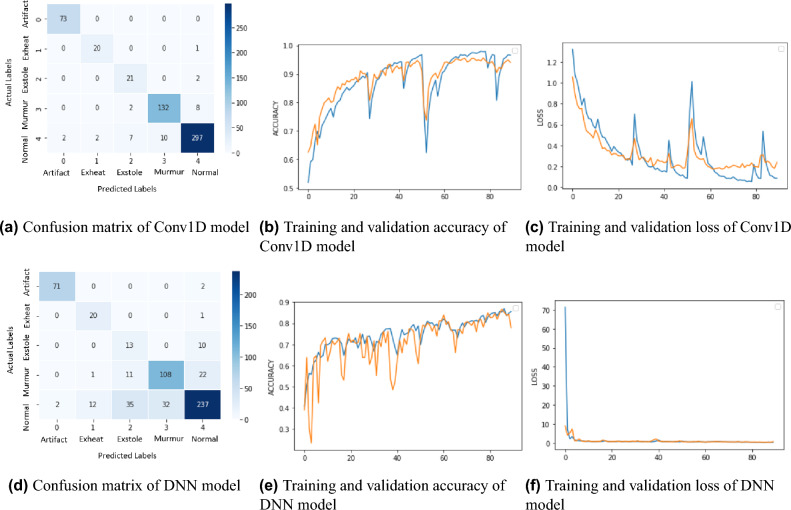
Visual representation of deep learning results.

This section explains the numerical outcomes corresponding to each deep learning model. The provided Table 6 displays the quantitative outcomes of two deep learning models, Conv1D and DNN. The metrics evaluated include precision, recall, F1-score, testing, and training accuracy. The performance metrics of each model will be individually elucidated, subsequently followed by a comparative analysis of the outcomes. The visual representation of deep learning model results is depicted in Fig. 8. The Conv1D model demonstrated a precision rate of 94.10%, a recall rate of 94.10%, and an F1-score of 94.06%. The aforementioned metrics indicate the model’s capacity to effectively classify various forms of heart disease. The Conv1D model exhibited a testing accuracy of 94.11%, indicating a commendable classification performance level across all categories. The observed discrepancy between the training accuracy, which recorded a higher value of 96.65%, and the testing accuracy implies a potential occurrence of overfitting, where the model may have excessively adapted to the training data.

**Table 6:** Deep Learning Numerical Results with Augmented Dataset.

Model	Training Accuracy (%)	Testing Accuracy (%)	Precision (%)	Recall (%)	F1-score (%)
Conv1D	96.65	94.11	94.10	94.10	94.06
DNN	85.54	78.20	82.09	78.50	79.10

The confusion matrix of the Conv1D model is depicted in Fig. 8a. The multiclass classification issue was modeled using a Conv1d model. 73 “artefact” samples were accurately identified as belonging to the “artefact” class. No instances of “artefact” being incorrectly categorized as another category (false positives) were found. Furthermore, no samples from other classes were incorrectly labeled as “artefact’ (false negatives). Twenty “exstole” samples were authentic (true positives), meaning the class was correctly identified. One “exstole” sample was incorrectly labeled as “normal” (a false positive). There were no “exstole” (false negative) samples found among the other categories. 21 of the “extrastole” samples were accurate predictions (true positives). Two “extrastole” samples were incorrectly labeled as “normal” (false positives). False negatives (samples incorrectly labeled “extrastole”) did not occur in other categories. 132 “murmur” samples could be confidently labeled as “murmur” (true positives). False-positive results (misclassification of “murmur” samples as “normal”) occurred 10 times. Also, two “murmur” samples were incorrectly labeled as “extrastole” (false positives). No samples from other categories were incorrectly labeled as “murmur” (false negatives). 297 “normal” samples could be confidently identified as “normal” (true positives). At least two “normal” samples were incorrectly labeled as “artefact” (false positives). False positives (classification of a “normal” sample as an “exstole” sample) occurred twice, as well. There were 7 false positives (samples incorrectly labeled as “extrastole”) among the “normal” group. Two “normal” samples were incorrectly labeled as “murmur” (false positives). Figure 8b,c show that the CNN model performed best in training and validation and had fewer losses, making it a more reliable tool for predicting heart disease on new data. These graphs depict the changes in training accuracy, validation accuracy, training, and validation loss during the training period. The training accuracy is depicted by the blue line in Fig. 8b, exhibiting a steady and continuous increase across the epochs. The initial value is roughly 51.93%, and it progressively rises, reaching around 96.66% after the 90 epochs. This indicates that the model efficiently acquires knowledge from the training data. The yellow line represents the validation accuracy, exhibiting a comparable pattern. The initial value is 62.56%, and it consistently rises, eventually reaching approximately 94.11%. The training and validation accuracy convergence suggests that the model effectively generalizes to unfamiliar input. The blue line in Fig. 8c represents the training loss, which constantly reduces from an initial value of 1.3187% to around 0.0860%. This shows that the model successfully minimizes its error on the training data. The yellow line depicts the validation loss, which begins at 1.0540% and reduces across epochs to approximately 0.2384%. The convergence of training and validation losses indicates that the model is not overfitting and works well on fresh, previously unseen data.

DNN model demonstrated a precision rate of 82.09%, a recall rate of 78.50%, and an F1-score of 79.10%. The metrics demonstrate a comparatively diminished precision, recall, and F1-score concerning the Conv1D model. The DNN model achieved a testing accuracy of 78.20%, suggesting a moderate level of classification performance across all categories. The training accuracy exhibited a value of 85.54%, indicating a certain overfitting level. The confusion matrix of the DNN model is depicted in Fig. 8d. 71 “artefact” samples were accurately identified as belonging to the “artefact” class. Two “artefact” samples were incorrectly identified as “normal” (false positives). False negatives (samples of other classes incorrectly labeled as “artefact”) did not occur. 20 “exstole” samples were authentic (true positives), meaning the class was correctly identified. Only one “exstole” sample was incorrectly labeled as “normal” (false positive). No “exstole” (false negative) samples were found among the other categories. 13 “extrastole” samples could be confidently identified as “extrastole” (true positives). Ten “extrastole” samples were incorrectly labeled as “normal” (10 false positives). False negatives (samples incorrectly labeled “extrastole”) did not occur in other categories. Correctly identified “murmur” samples (i.e., “true positives”) numbered 108. One “murmur” sample was incorrectly labeled as “exstole” (a false positive). False positives (misclassifications of “murmur” samples as “extrastole”) occurred in 11 cases. False positives (classifying “murmur” samples as “normal”) occurred 22 times. In the “normal” category, 237 samples were correctly identified as such (true positives). There were four false positives (samples incorrectly labeled as “artefact”) that originated from “normal” conditions. A further 12 “normal” samples were incorrectly labeled as “exstole” (false positives). There were 35 false positives (samples incorrectly labeled as “extrastole”) among the “normal” group. In total, 32 “normal” samples were incorrectly labeled as “murmur” (false positives). Elements of the confusion matrix that fall on the diagonal reflect correctly categorized samples (true positives) for each class. In contrast, those that fall off the diagonal represent incorrectly classed samples (false positives).

Figure 8e,f show that the DNN model achieved a normal accuracy score during training and validation. Still, it also displayed considerable loss, making it a significant challenge for the DNN model to predict heart disease on new data. The blue line in Fig. 8e represents the training accuracy, which increases throughout the training procedure. Initially at 40.65% in Epoch 1, the accuracy gradually increases over the following epochs, reaching around 85.55% by the end of Epoch 90. In contrast, the validation accuracy, represented by the yellow line, starts at 38.99% in the first epoch and gradually increases. However, about Epoch 30, it appears to plateau with occasional variations. Finally, by Epoch 90, the validation accuracy has stabilized at roughly 77.82%. The obvious difference between training and validation accuracies in the latter epochs indicates the onset of probable overfitting, needing more analysis and model changes. The training loss, represented by the blue line in Fig. 8f, has decreased significantly from the high value of 71.3196 in the first epoch. This falling pattern continues into successive epochs, albeit at a slower reduction rate. By the end of Epoch 90, the training loss is a reasonably low value of 0.3651. The validation loss, illustrated by the yellow line, follows a similar course, beginning at 8.9633 and rapidly dropping initially. However, around Epoch 40, a substantial increase indicates a possible overfitting problem. The validation loss reaches 0.6353 by the end of the training period. The discernible discrepancy between training and validation losses emphasizes the importance of carefully considering regularization strategies or early stopping to optimize the model’s generalization to new inputs.

When comparing the outcomes of the models, it was observed that the Conv1D model consistently exhibited superior performance compared to the DNN model across all evaluation metrics. The Conv1D model exhibited superior precision, recall, F1-score, testing, and training accuracy. The Conv1D model’s superior performance can be attributed to its capacity to effectively capture temporal patterns and dependencies in audio signals. This characteristic proves advantageous when analyzing sequential data, such as heart sounds. The disparity between training accuracy and testing accuracy can be ascribed to the phenomenon of overfitting. Overfitting is a phenomenon that arises when a model becomes excessively tailored to the specific characteristics of the training data, resulting in a diminished ability to generalize its predictions to new, unseen data effectively. In this scenario, the elevated training accuracy implies that the models have likely achieved high proficiency in fitting the training data. However, their performance on novel, unseen data may be less effective, leading to a slightly diminished testing accuracy. To mitigate the issue of overfitting and enhance the efficacy of the models, it is advisable to explore techniques such as regularization, adjusting the complexity of the model, or augmenting the volume of training data.

### Experimental results comparison of original and augmented dataset

When the findings of the original dataset from Table 4 are compared to those of the augmented dataset as shown in Tables 5 and Table 6, significant variations in model performance are shown. DT is the top performing machine learning model using the original dataset, with accuracy, precision, recall, and F1-scores of roughly 77.77%, 78.10%, 78.10%, and 78.10%, respectively. K-Nearest Neighbours (KNN) fared well as well, with an accuracy of 68.88% and precision, recall, and F1-score values of 69.10%. Conv1D and DNN deep learning models, on the other hand, achieved lower accuracy and F1-score values, with Conv1D at 53.64% and 38.60% and DNN at 54.10% and 38.10%, respectively. However, when the augmented dataset is used, there is a significant improvement in model performance across the board. RF, MLP, and XGB models have significantly improved accuracy, precision, recall, and F1-score. MLP and XGB achieved astounding accuracy levels of 95.65% and 95.31%, respectively, with precision, recall, and F1-score values over 96%. Random Forest achieved an accuracy of 80.92% with precision, recall, and F1-score values that were balanced at around 81.89%. KNN and DT also improved performance. With the augmented dataset, both Conv1D and DNN deep learning models showed significant improvements in all measures. Conv1D achieved 94.10% precision, recall, and F1-score, while DNN achieved 82.09% precision, recall, and F1-score, respectively.

Overall, the augmented dataset significantly increased the performance of all models, with Random Forest, Multilayer Perceptron, and XGBoost emerging as the machine learning category’s best performers. Conv1D significantly improved its performance in the deep learning area, although it still lags behind machine learning models in other criteria. The original dataset results were significantly poorer overall performance, except for Decision Tree and K-Nearest Neighbours in the machine learning category.

### Comparison with existing studies

Table 7 presents a comprehensive comparison of various methodologies currently employed in the classification of heart sound signals to detect distinct cardiac diseases, namely “normal,” “murmur,” “extra heart sound,” “artefact,” and “extrastole.” Every row in the table corresponds to a distinct reference or study employing diverse classification models and features. Let us proceed with a detailed analysis of the provided information. The study ^41^ employed a CNN that incorporated Wavelet-based Features to classify heart sound signals. An accuracy rate of 82.22% was achieved during the testing process. The present study^11^ utilized SVM in conjunction with MFCC features for classification. The testing accuracy achieved was 85.36%. The study^42^ employed the KNN algorithm in conjunction with MFCC as features for classification. The resulting testing accuracy was determined to be 84.53%. Heart sound signal classification was performed in a study^43^ using a CNN in conjunction with MFSC features. The testing accuracy achieved was 88.18%. In the study^8^, a different CNN architecture was employed, utilizing MFSC as input features. This alternative model demonstrated improved performance, achieving a testing accuracy of 93.88%. The aforementioned approach signifies the novel methodology under consideration in the present investigation. The classification model employed in this study is an MLP that utilizes a Feature Vector consisting of MFCC features. The model under consideration attained the highest level of testing accuracy compared to all the presented techniques, achieving a score of 95.65%. The proposed model’s precision, defined as the ratio of real positive predictions to total predicted positives, is claimed to be 96.60%. This implies that when the model predicts a favorable outcome, it is 96.60% accurate. Furthermore, the proposed model has a recall of 97.60%. The model’s ability to capture all relevant class occurrences is reflected in recall, also known as sensitivity or true positive rate. With a recall of 97.60%, the model correctly detects many real positive events. The proposed model has an F1-score of 96.60%. The F1-score compromises precision and recall, thoroughly evaluating the model’s overall efficacy. The previous research only focused on testing accuracy, while this research utilized multiple evaluation metrics to evaluate the model better.

The findings indicate that an MLP model utilizing the MFCC as feature performs better than current methodologies in testing accuracy. This outcome positions the proposed approach as promising for classifying heart sound signals and detecting various heart diseases. It is imperative to acknowledge that the selection of features, algorithms, and architectures can substantially influence the performance of the classification model. The method proposed in this study has demonstrated the highest level of accuracy, suggesting its potential for effectively and dependably classifying heart disease. However, additional validation and comparison using larger datasets, and potentially across diverse datasets, would be required to ascertain the generalizability and robustness of the proposed methodology.

**Table 7:** Comparison with existing techniques.

Ref.	Model/Features	Testing Accuracy	Precision	Recall	F1-score
^41^	CNN with Wavelet-based Features	82.22%	–	–	–
^11^	SVM with MFCC Features	85.36%	–	–	–
^42^	KNN with MFCC Features	84.53%	–	–	–
^43^	CNN with MFSC Features	88.18%	–	–	–
^8^	CNN with MFSC Features	93.88%	–	–	–
Proposed	MLP with MFCC Features Vector	95.65%	96.60%	97.60%	96.60%

## Conclusion

The need for reliable and accurate methods of diagnosing cardiovascular disorders is growing. Early diagnosis is crucial for improving patient outcomes due to the prevalence of CVD worldwide. The application of ML and DL techniques has been successful in classifying heart sounds. The signals were processed, sampled, and graphically represented using spectrograms and MFCCs in this work, which used the PASCAL CHALLENGE database. This research presents an efficient approach based on machine learning and deep learning techniques to detect heart disease from noisy sound signals. First, data augmentation has added synthetic noise to the heart sound signals. Secondly, the feature ensembler has been developed by combining the features of multiple audio feature extraction techniques. In the end, several machine learning and deep learning models were employed to detect heart disease. Deep learning models include Conv1D and DNN, whereas machine learning models include RF, MLP, XGB, KNN, and DT. When the outcomes of the models employed in this study were compared, the MLP model produced the best results, with an accuracy of 95.65% and a *P*-value of 1.0 decimals.

*Future Work:* Several areas for future research can be explored to improve heart disease detection using noisy sound sources. More advanced noise augmentation approaches could be developed to imitate real-world scenarios better and boost the model’s robustness. Furthermore, investigating advanced feature ensembling approaches such as deep feature fusion or attention mechanisms may improve the model’s ability to acquire meaningful information from varied feature sets. Furthermore, research into transfer learning methodologies that use pre-trained models on big audio datasets to improve performance on heart sound signals could be advantageous. Incorporating domain knowledge and expert annotations into the model may also increase interpretability and therapeutic relevance. This research aims to identify heart disease from noisy audio signals; although computational cost is one of the main parameters, it was not considered in this instance. This research recognizes its significance for future work.

Furthermore, experimenting with alternate architectures and hyperparameter tuning for deep learning models may result in additional performance gains. Finally, larger and more diverse datasets might be used to test the model’s generalizability and possible applicability in real-world clinical settings. These future initiatives are intended to improve the accuracy and reliability of cardiac disease detection, resulting in more effective and prompt medical interventions. We made a deliberate decision not to address data imbalance in the current phase of our research. This decision was made because resolving data imbalance in the context of audio data is a critical and difficult undertaking. It necessitates considerable consideration and sophisticated approaches that require undivided attention. However, we understand the importance of fixing data imbalance and that it is our top priority. In the future, we want to address this issue thoroughly. We will employ strategies and ways to deal with data imbalance while maintaining the integrity and quality of our study results. This approach ensures that our study remains thorough and accurate while addressing audio data imbalance.

## Data Availability

The datasets analyzed during the current study are available in the Kaggle repository, [https://k4all.org/2011/11/announcing-the-pascal-heart-sounds-challenge/].
